# Effect of exogenous circulating anti-bPL antibodies on bovine placental lactogen measurements in foetal samples

**DOI:** 10.1186/1751-0147-52-9

**Published:** 2010-02-03

**Authors:** Andrea Vivian Alvarez-Oxiley, Noelita Melo de Sousa, Jean-Luc Hornick, Kamal Touati, Gysbert C van der Weijden, Marcel AM Taverne, Otto Szenci, Jean-François Beckers

**Affiliations:** 1 Laboratory of Endocrinology and Animal Reproduction, Faculty of Veterinary Medicine, University of Liege, Belgium; 2 Nutrition of Large Animals, Faculty of Veterinary Medicine, University of Liege, Belgium; 3 Clinic of Large Animals, Faculty of Veterinary Medicine, University of Liege, Belgium; 4 Department of Farm Animal Health, Faculty of Veterinary Medicine, Utrecht, the Netherlands; 5 Clinic for Large Animals, Faculty of Veterinary Science, Szent Istvan University, Budapest, Hungary

## Abstract

**Background:**

The involvement of placental lactogen (PL) in the regulation of foetal growth has been investigated in different species by *in vivo *immunomodulation techniques. However, when circulating antibodies are present together with the hormone, the procedure for hormonal measurement becomes considerably complex. The aim of this study was the immunoneutralization of bovine placental lactogen (bPL) concentrations in bovine foetal circulation by direct infusion of rabbit anti-bPL purified immunoglobulins (IgG) via a foetal catheter (*in vivo *study). The ability of a RIA based on guinea pig anti-bPL antiserum, for the measurement of bPL concentrations in samples containing exogenous rabbit anti-bPL immunoglobulins, was also analyzed in *in vitro *and *in vivo *conditions.

**Methods:**

Six bovine foetuses were chronic cannulated on the aorta via the medial tarsal artery. Infusion of rabbit anti-bPL IgG was performed during late gestation. Pooled rabbit anti-bPL antisera had a maximal neutralization capacity of 25 μg bPL/mL of immunoglobulin. Interference of rabbit anti-bPL immunoglobulin with radioimmunoassay measurement using guinea pig anti-bPL as primary antibody was first evaluated *in vitro*. Polyclonal anti-bPL antibodies raised in rabbit were added in foetal sera to produce 100 samples with known antibodies titers (dilutions ranging from 1:2,500 till 1:1,280,000).

**Result(s):**

Assessment of the interference of rabbit anti-bPL antibody showed that bPL concentrations were significantly lower (P < 0.05) in samples added with dilutions of rabbit antiserum lower than 1:80,000 (one foetus) or 1:10,000 (four foetuses). It was also shown that the recovery of added bPL (12 ng/mL) was markedly reduced in those samples in which exogenous rabbit anti-bPL were added at dilutions lower than 1:20,000. Concentrations of foetal bPL were determined in samples from cannulated foetuses. In foetuses 1 and 6, bPL concentrations remained almost unchanged (<5 ng/mL) during the whole experimental period. In Foetus 3, bPL concentrations decreased immediately after IgG infusion and thereafter, they increased until parturition.

**Conclusion(s):**

The use of a bPL RIA using a guinea pig anti-bPL as primary antiserum allowed for the measurement of bPL concentrations in foetal plasma in presence of rabbit anti-bPL IgG into the foetal circulation. Long-term foetal catheterization allowed for the study of the influence of direct infusion of anti-bPL IgG on peripheral bPL concentrations in bovine foetuses.

## Introduction

Growth hormone (GH), prolactin (PRL), and placental lactogen (PL) are members of a family of polypeptide hormones that are thought to have arisen from a common ancestral gene. GH and PRL are mainly secreted by the anterior pituitary of all vertebrates, whereas PL is uniquely observed in some mammalian species and is secreted in the placenta by trophoblastic cells. PL shares several structural and biological activities with GH and PRL. As reviewed by Goffin et al. [1], classically, the GH receptor (GHR) was presented as the specific receptor for GH, whereas the PRL receptor (PRLR) was considered specific for PRL and PL. It has been also shown that both bovine (b) and ovine (o) PL can bind to GHR [2,3]. The involvement of PL in the regulation of foetal growth has been investigated in different species. In human (h), hPL might be a foetal somatogenic hormone as suggested by the presence of specific hPL receptors in foetal tissues and by the fact that hPL but not hGH can stimulate amino acid uptake and glycogenesis in foetal tissues [4]. The results from studies in ruminant species in which PL levels were altered by infusion of PL molecules into the maternal and foetal circulations [5-7] have also suggested that PL regulates foetal growth by stimulating uptake of maternal nutrients to the foetus and by stimulating the foetus to use the substrates.

Immunoneutralization of different hormones such as ovine PL [8] and somatostatin [9] have also been conducted in order to investigate endocrine growth pathways *in vivo*. However, when circulating antibodies are present together with the hormone, the procedure for hormonal measurement becomes considerably complex. Different methods have been proposed to detect and to eliminate this interference in radioimmunoassay (RIA) systems [10]. These include serial dilutions of the sample [11], polyethylene glycol precipitation [12], blocking with nonimmune serum [13] and use of alternative antibodies reacting with epitopes and believed to be distinct from those recognized by circulating antibodies [14].

Recently, we have performed foetal cannulation in bovine species in order to investigate the effect of immunoneutralization of bovine placental lactogen (bPL) on some hormonal parameters assumed to be related to foetal growth [15]. Bovine PL binds both somatogenic and lactogenic receptors with high affinity [16]. In bovine species, PL concentrations have a very particular distribution in maternal and foetal compartments. Maternal concentrations remain under 2 ng/mL during the whole pregnancy period, whereas foetal concentrations are higher, ranging from 25 to 30 ng/mL on Day 90 of gestation and decreasing to 5-15 ng/mL near term [17]. Despite all the knowledge generated to date, the biological activity of bPL in foetal growth remains largely unknown [18]. The placental origin of this hormone [19] and the repartition of the hormone mainly in the foetal circulation than in the maternal one constitute major difficulties for *in vivo *investigations.

We designed the present study in order to analyze the ability of a RIA based on guinea pig anti-bPL antiserum for the measurement of bPL concentrations in foetal samples containing exogenous rabbit anti-bPL antisera under *in vitro *and *in vivo *conditions.

## Materials and methods

### Reagents

Most of chemical reagents used for RIA were purchased from Merck (Darmstadt, Germany) with the exception of sodium azide (NaN_3_; Vel, Leuven, Belgium), bovine serum albumin (BSA Fraction V; ICN Biochemicals Inc., Aurora, OH), detergent polysorbate (Tween 20™; Fluka, Buchs, Switzerland), and polyethylene glycol 6000 (Vel). Sephadex G-75 as well as ^125^I-Na were obtained from Amersham Biosciences (Uppsala, Sweden). Lactoperoxidase was purchased from Boehringer Ingelheim GmbH Corp. (Ingelheim, Germany). Native glycosylated 33 kDa form of bPL (nbPL; fraction 322), which was used as the standard was purified in our laboratory (Laboratory of Animal Endocrinology and Reproduction, University of Liege). Recombinant bovine placental lactogen (rbPL) used for radiolabeling was kindly provided by Dr. Parlow (rbPL, Lot#AFP9152C; NHPP, NIDDK & Dr. Parlow, USA).

### Origin of anti-bPL antibodies

Polyclonal antisera (AS) used for RIA were raised in guinea pig (AS#276) and rabbit (AS#295) against a highly purified bPL preparation (33 kDa) [20] according to the method of Vaitukaitis et al. [21]. The immunization protocol was approved by the Animal Ethics Committee of the University of Liege (Dossier number 287).

Optimal dilution titers (20 to 30% binding ratio of the radiolabeled rbPL (^125^I-rbPL) to the antiserum in the zero standard (B_0_) assay tube) were 1:130,000 for guinea pig AS#276 and 1:400,000 for rabbit AS#295 [22].

For the infusion proposal (*in vivo *study), an immunoglobulin (Ig) preparation was purified from a pool of rabbit anti-bPL antisera (AS#277, AS#278, AS#282, AS#284, AS#285, AS#286, AS#288, AS#289, AS#294, and AS#296, 700 mL) by using the method previously described by Harboe and Ingild [23]. The purified preparation (containing rabbit IgG anti-bPL) was ultrafiltered in an Amicon Cell System (MW 10,000 Da cut-off membranes) to reach a concentration of 5 mg Ig/mL, as determined by Lowry's method [24]. The purified Ig was extensively dialyzed against 0.9% NaCl (4 baths of 20 liters, 4°C) and stored at -20°C until use.

### Secondary antibodies used in double-antibody precipitation systems

Rabbit anti-guinea pig and sheep anti-rabbit secondary antibodies were obtained following the immunization protocol of Vaitukaitis et al. [21].

Specificity of secondary antibodies was tested by adding them to different samples containing primary guinea pig or rabbit antibodies. In brief, 100 μL of guinea pig AS#276 (1:130,000), rabbit AS#295 anti-bPL (1:400,000), or a mixture of both primary antisera (0.05:0.05 mL; vol:vol) were incubated with 100 μL of ^125^I-rbPL (25,000 cpm) [22]. The volume was adjusted to 500 μL by adding 300 μL of assay buffer (phosphate 0.05 M, pH 7.3 containing 0.1% BSA). After 24 h, 1 mL of PEG solution containing 0.87% v:v sheep anti-rabbit Ig or 0.45% v:v rabbit anti-guinea pig Ig were added to those tubes containing guinea pig AS#276 and rabbit AS#295, respectively. A further incubation (1 h 30 min) was realized at room temperature (20 to 25°C). The tubes were then washed with 2 mL of assay buffer containing 0.5% Tween 20™ and centrifuged at 2,500 × *g *at 4°C for 30 min. The supernatant was discarded and the pellet was counted in a gammacounter (LKB Wallac 126 multigamma counter, Turku, Finland) with a counting efficiency of 75%.

### Measurement of binding ratio of the anti-bPL antiserum to the tracer

Binding ratio of anti-bPL antiserum to the tracer (B/T, %) was measured in all bovine foetal samples. Briefly, 10 μL of each sample and 100 μL of ^125^I-rbPL (25,000 cpm) were added in tubes containing 400 μL of assay buffer. Samples were incubated overnight at room temperature. The next day, bound and free fractions were separated after addition of 1 mL of second-antibody PEG solution containing 0.87% v:v sheep anti-rabbit Ig, as described elsewhere [22].

### Bovine PL measurement in foetal samples

Concentrations of bPL in bovine foetal samples were measured by a double-antibody-binding RIA system. In brief, duplicate aliquots of foetal samples (50 μL) and 100 μL of each point of nbPL standard curve (100, 50, 25, 12.5, 6.25, 3.12, 1.56, and 0.78 ng/mL) were dispensed into conical tubes containing 300 μL of assay buffer, then incubated with 100 μL of ^125^I-rbPL (25,000 cpm) and 100 μL of diluted primary antibody (guinea pig anti-bPL). Initial dilution of the antiserum was 1:130,000. The maximum binding (B_0_) was determined by replacing standard nbPL by 100 μL of assay buffer. The nonspecific binding (NSB) tubes contained 400 μL of assay buffer and 100 μL of ^125^I-rbPL. Total count tubes (Tc) contained 100 μL of ^125^I-rbPL. The following day, for separation of bound and free fractions, 1 mL of second-antibody PEG solution (0.05% v:v normal guinea pig serum, 0.45% v:v rabbit anti-guinea pig antiserum, 0.4% w:v BSA, 0.05% w:v microcrystalline cellulose, 0.5% w:v polyethylene glycol 6000 in phosphate buffer) was added to all except the Tc tubes and a further incubation (1 h 30 min) was realized at room temperature. The tubes were then washed with 2 mL of phosphate-BSA-Tween 20™ buffer and centrifuged at 2,500 × g at 4°C for 30 min. The supernatant was discarded and the pellet was washed again. The radioactivity was measured in a gammacounter with an efficiency of 75%.

The minimal detection limit (MDL) was determined as the mean concentration minus twice the standard deviation of 20 replicates of the zero standard. Four plasma samples with distinct bPL concentrations were used to calculate intra-assay and inter assay variations.

### *In vitro *study on foetal samples containing anti-bPL exogenous antibody

Interference of exogenous rabbit (AS#295) anti-bPL primary antiserum with *in vitro *measurement of bPL concentrations was analyzed by adding different dilutions of this antiserum to 5 bovine foetal samples containing the following amounts of bPL: 8.9 ± 1.6 ng/mL (Foetus A), 10.0 ± 1.3 ng/mL (Foetus B), 17.5 ± 1.4 ng/mL (Foetus C), 18.5 ± 1.9 ng/mL (Foetus D) and 21.3 ± 1.5 ng/mL (Foetus E). The samples were collected at a slaughterhouse from 90- to 280-days-old bovine foetuses. The foetal ages were determined by crown-rump measurement [25]. Serum was allowed to clot, centrifuged (15 min at 1,500 × g), aliquoted, and stored at -20°C until use.

In brief, 50 different stock solutions were prepared by adding 100 μL of different dilutions of rabbit AS#295 (1:500 to 1:256,000) to 400 μL of each foetal sample. Foetal samples were pre-incubated with diluted antiserum for 10 h (room temperature) before RIA analysis. The final dilutions of rabbit antiserum ranged from 1:2,500 to 1:1,280,000.

A recovery test was carried out by adding to each foetal sample (70 μL) 30 μL of phosphate-BSA buffer containing 40 ng/mL of bPL to obtain a final concentration of 12 ng/mL. Recoveries of bPL were calculated as the observed/expected bPL concentrations. The final results were expressed as the percent recovery of each tested sample.

### *In vivo *study in cannulated bovine foetuses

**Figure 1:**
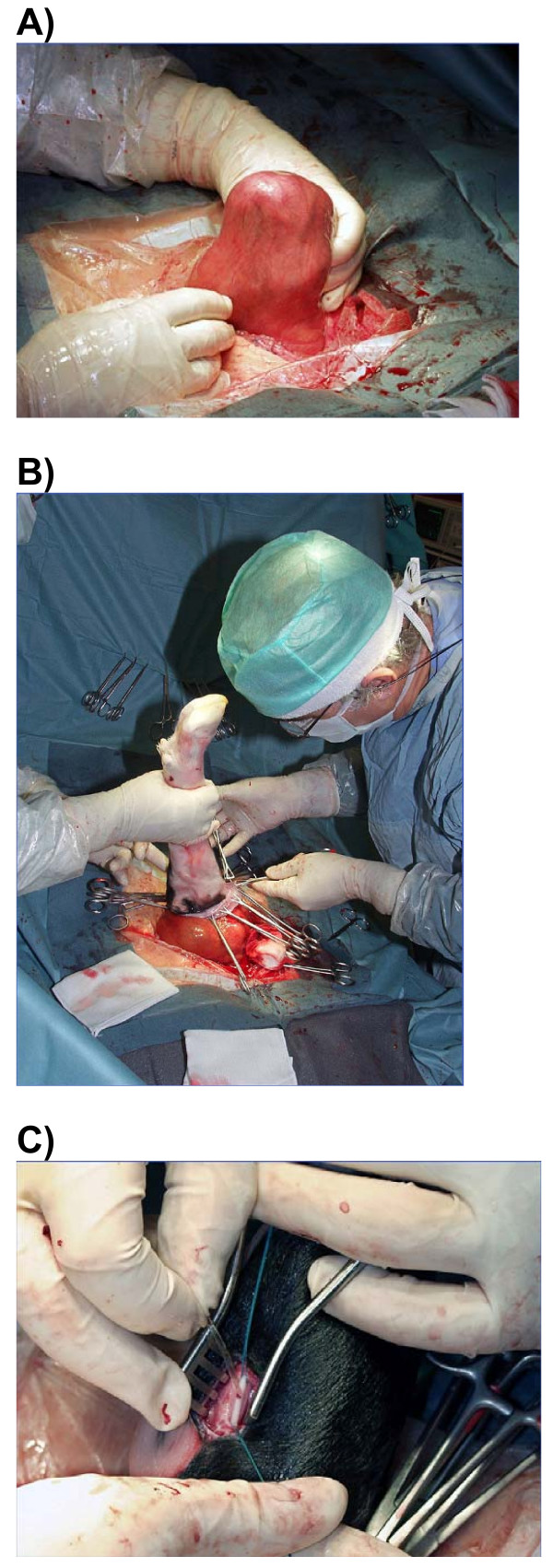
**Arterial cannulation in bovine foetuses**. Bovine fetal hind limb was identified by intra-abdominal palpation and moved so that the foot lay in the maternal abdominal incision (A). After an incision through the uterine wall and after opening of the fetal membranes, they were progressively fixed together by Collins forceps. The fetal limb was withdrawn from the uterus until the anterior surface of the hock joint was easily accessible (B). The fetal medial tarsal artery was exteriorized and catheterized with a polyvinyl catheter (C).

Six Holstein pregnant cows were used for this study. The experimental protocol was approved by the ULg Ethics Committee (Dossier number 125). Gestational age on the day of surgery varied from approximately 180 days (6 months) to 249 days (8 months) post-insemination. The cannulation of the medial tarsal artery (polyvinyl catheter, 0.75 mm I.D × 1.45 mm O.D) was based on the technique previously described by Taverne et al. [26] with some modifications. In brief, following general anesthesia with halothane and surgical preparation, the uterus was exposed through a median incision on linea alba. The foetal hind limb was identified by intra-abdominal palpation and moved so that the foot could be presented in the abdominal incision (Figure 1A). After an incision through the uterine wall, foetal membranes were successively incised and progressively fixed together by Collins forceps (Figure 1B). The foetal limb was withdrawn from the uterus until the anterior surface of the hock joint was easily accessible. Care was taken to keep the loss of foetal fluids to a minimum. The foetal medial tarsal artery was exteriorized and catheterized with a polyvinyl catheter (Figure 1C). The catheter was advanced 40-50 cm so as to lie in the dorsal aorta. And then, the foetal catheter was fixed to the skin, and after foetal tissues closure, the foetal leg was carefully returned to the uterus. Approximately 50 to 60 cm of catheter were inserted into the uterine cavity. The uterus was then closed with two rows of continuous sutures (simple and Cushing) for foetal membranes and the uterine wall. Before the mid-ventral skin was sutured, the free extremity of the catheter was exteriorized through a small incision on the left side of the abdominal wall. The abdominal midline incision was closed using a three-layer suture standard procedure. The catheter was tunneled subcutaneously along the flank to the most dorsal area of the left sublumbar fossa. Hypodermic blind needles capped with Luer-lock injection caps were inserted into the external end of the catheter. The catheter was filled with 5 mL of a sterile heparinized saline solution (0.9% NaCl containing 200 units of heparin/mL) and kept into a plastic bag containing a 50:50 v:v ethanol:distilled water solution.

In the morning following surgery, each cow was placed in a pen where she remained until calving. Cows were fed with grass hay twice a day and water was available at all times. The external ends of the catheters were transferred into a hood containing a small container filled with 50% ethanol solution. The catheter was flushed with 3 to 5 mL of sterile heparinized saline (200 units of heparin/mL) once daily until parturition.

Heparinized blood samples (3 mL) were taken from foetuses by using strict aseptic procedures. Sampling of foetal blood was begun on the fourth day after cannulation and was performed on a daily basis, usually between 8.00 and 12.00 a.m., until parturition. In most cases, foetal samples could be obtained. However, in some days, samples could not be withdrawn probably due to the interference of a blood clot with the catheter or due to the positioning of the foetal leg. After each sampling, catheters were flushed and filled with 5 mL of heparinized saline. All the collected samples were immediately centrifuged at 1,500 × *g *(4°C) during 15 min. Plasma was aliquoted in small volumes (500 μL) and stored at -20°C until assayed for bPL as previously described.

Infusion of rabbit anti-bPL IgG into the foetal circulation begun on Days 6 to 14 after surgery. Table 1 describes the period of pregnancy, the volume and the frequency of infusion of IgG anti-bPL in bovine foetal circulation. In order to avoid any foetal contamination, the IgG solution was filtered in a 0.2 μm sterile acrodisc filter (Pall Life Sciences, Cornwall, United Kingdom) immediately before injection.

**Table 1:** Days and doses of immunoglobulins infused into the foetal circulation of cannulated foetuses.

Foetus	Infusion		IgG anti-bPL infused per Day	
	
	Day of pregnancy	Day after surgery	Volume	mg of IgG
Foetus 1	238	6	1 × 8 mL	40 mg
Foetus 2	239	6^a^	2 × 10 mL	100 mg
Foetus 3	249	14^a^	2 × 10 mL	100 mg
Foetus 4	256	7^a^	2 × 10 mL	100 mg
	262 and 263	13^a ^and 14^a^	2 × 10 mL	100 mg
	271 to 276	22^a ^to 27^a^	2 × 10 mL	100 mg
Foetus 5	243	6^a^	2 × 10 mL	100 mg
	258	21^a^	2 × 10 mL	100 mg
Foetus 6	≈ 6 months	8	1 × 4 mL	20 mg
		20	1 × 8 mL	40 mg
		41^a^	2 × 10 mL	100 mg
		61^a ^and 62^a^	2 × 10 mL	100 mg
		85^a^	2 × 10 mL	100 mg

Immunoglobulins were raised in rabbits against a glycosylated native form of bovine placental lactogen (nbPL).

^a^Infusion twice daily (interval between two consecutive infusions on the same day varied from 9 to 12 h).

### Statistical analysis

Descriptive data are shown as the mean of values obtained from the experiments performed in duplicate by using Statview program [27]. Statistical significance was accepted at the *P *< 0.05 level.

The effects of antisera dilutions on bPL concentration measurements (*in vitro *study) were analyzed using a general linear model (Proc GLM, SAS) according to the following model: Yij = ai + bj + eij, where Yij = difference in bPL concentration measured in control sample and sample that received antisera, in animal i (i = 1 to 5) and at dilution j (1:1,280,000 to 1:2,500 step 2 dilution), ai = the effect of animal i, bj = effect of the dilution j, and eij is the random residual effect (N [22]). The animal effect was considered as random and the dilution one as fixed. The random intra-treatment variance in control samples (samples which did not receive antisera) was considered to over-estimate the real value of the random residual variance. Thus, the effect of the treatment was finally tested on the difference between residual variance and 2 times the mean variance associated with the intra-treatment variability in control samples. The ratio of the mean delta obtained at each dilution level to this estimated residual variance was tested with a student *t*-test for 4 degrees of freedom (5 animals -1).

A similar model was used for data relative to recovery test, but the effect of treatment was simply tested on residual variance owing to the fact that no blank control was tested in this trial.

For the *in vivo *study, only bPL foetal profiles were described. Not all data were available for every animal at each time-point, largely because of failures in taking samples from the catheters.

## Results

### Characteristics of RIA used for bPL measurement in foetal samples

By using guinea pig anti-bPL antiserum, displacement of the standard inhibition curve ranged from 98 to 13% of binding (B/B_0_). The minimum concentration of bPL detected by this RIA system was 0.02 ng/mL. The bovine foetal samples showed parallel displacement to the standard curves (data not shown). Nonspecific binding was 1%. The intra-assay coefficients of variation at bPL concentrations of 14.0, 8.5, 5.5, and 1.6 ng/mL were 5.2, 5.4, 6.4, and 9.8%, respectively. Inter-assay coefficients of variation measured in the same samples were 9.6, 8.6, 7.8, and 11.0%, respectively.

### Specificity of secondary antibodies

As shown in Table 2, rabbit anti-guinea pig antisera did not precipitate the complex formed by ^125^I-rbPL and rabbit anti-bPL primary antiserum. By contrast, sheep anti-rabbit antisera were able to precipitate the guinea pig complex formed by ^125^I-rbPL and the guinea pig anti-bPL antiserum.

**Table 2:** Binding of primary rabbit (AS#295) or guinea pig (AS#276) antisera raised against glycosylated native form of bovine placental lactogen (nbPL) to the different secondary antisera.

Secondary antisera	Primary antisera	B/T	Cross reactivity
Rabbit anti-guinea pig	Rabbit AS#295	0.8%	-
	Guinea pig AS#276	12.7%	+
	AS#276 + AS#295	12.5%	+
Sheep anti-rabbit	Rabbit AS#295	12.2%	+
	Guinea pig AS#276	8.0%	+
	AS#276 + AS#295	8.3%	+

B/T: Binding activity (B) regarding added Tracer (T).

### Measurements of foetal bPL in the presence of exogenous anti-bPL antibodies (*in vitro *study)

Foetal concentrations of bPL were measured in the presence or absence of exogenous rabbit antibodies by using guinea pig primary antiserum. The concentrations of bPL before the addition of the antiserum ranged from 6.7 (Foetus A) to 22.6 ng/mL (Foetus 6). The binding activity measured as B/T (%) ranged from 3 to 27% in samples containing anti-bPL dilutions ranging from 1:1,280,000 to 1:2,500, respectively. Despite the use of a guinea pig RIA system, in Foetus A, which gave the lowest bPL levels, concentrations decreased by more than 50% when an antiserum dilution of 1:80,000 was added. For the other foetuses, concentrations lower than 50% of the initial bPL concentrations (sample before antiserum addition) were observed when rabbit anti-bPL sera were added at dilutions of 1:10,000 to 1:2,500.

The percentages of recovery of bPL in the presence of exogenous rabbit anti-bPL in those foetal samples having been added of 12 ng/mL of bPL were shown in Table 3. Samples containing lower dilutions of rabbit anti-bPL sera were not quantified exactly. The recovery ranged from 42 to 48% at a rabbit anti-bPL dilution of 1: 2,500. The accuracy of the measurement showed a significant increase (*P *< 0.05) when rabbit anti-bPL dilutions were equal to or higher than 1: 20,000 (recovery higher than 70%).

**Table 3:** Recovery of 12 ng/mL of bPL added to five foetal samples (A to E) in the presence of different dilutions of rabbit anti-bPL.

Initial serum: bPL (ng/mL)	Identification of foetal sample	Foetal bPL concentrations (ng mL^-1^) and percentage of recovery					
		Rabbit anti-bPL dilutions					
		1:1,280,000	1:320,000	1:80,000	1:20,000	1:5,000	1:2,500
A: 8.9 ± 1.6	A+	8.6	7.5	3.7	2.6	1.6	0.7
	A*	20.6	19.5	15.7	14.6	13.6	12.7
	A++	20.5	19.0	13.9	11.3	6.9	5.4
	Recovery (%)	99.4	97.4	88.3	77.3	50.4	42.9
							
B: 10.0 ± 1.3	B+	11.5	7.6	6.3	6.0	1.5	0.7
	B*	23.5	19.6	18.3	18.0	13.5	12.7
	B++	23.9	18.4	17.2	14.9	8.1	6.3
	Recovery (%)	101.7	93.9	93.9	82.7	60.2	49.1
							
C: 17.5 ± 1.4	C+	18.1	19.4	15.4	16.4	3.3	0.9
	C*	30.1	31.4	27.4	28.4	15.3	12.9
	C++	29.5	28.5	23.8	20.7	10.7	5.3
	Recovery (%)	98.1	90.8	87.1	72.8	70.1	41.4
							
D: 18.5 ± 1.9	D+	20.3	21.0	14.6	12.4	6.6	0.9
	D*	32.3	33.0	26.6	24.4	18.6	12.9
	D++	31.5	30.9	24.4	19.5	10.5	5.9
	Recovery (%)	97.3	93.7	91.6	80.0	56.5	45.7
							
E: 21.3 ± 1.5	E+	22.56	19.3	21.2	15.3	5.2	1.2
	E*	34.56	31.3	33.2	27.3	17.2	13.2
	E++	35.60	28.8	30.6	20.2	11.2	6.3
	Recovery (%)	103.01	92.2	92.2	74.1	64.8	47.7

+: Concentrations of bPL in the presence of different dilutions of rabbit anti-bPL;

*: Theoretical bPL concentrations after addition of 12 ng/mL of bPL;

++: Observed bPL concentration.

### Measurement of foetal bPL in the presence of circulating anti-bPL antibodies throughout late gestation

**Figure 2:**
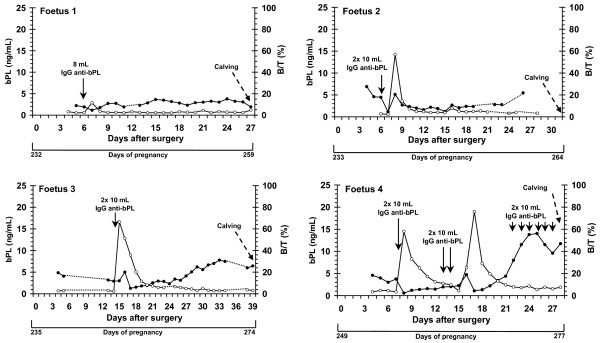
**Plasmatic profiles of bPL concentrations and rabbit anti-bPL titers in peripheral circulation of four bovine foetuses**. Concentrations of fetal bPL (ng/mL) are represented by black dots. Rabbit anti-bPL titers measured as B/T (bound activity (B) regarding total tracer (T) added) are represented by white circles. Plasma samples from cannulated foetuses (Foetuses 1 to 4) were collected from Days 232 (Foetus 1) to 249 (Foetus 4) of pregnancy until term. Concentrations of bPL were measured by RIA with guinea pig anti-bPL antiserum (AS#276) as primary antibody. Solid line arrows indicate day of infusion of a pool of rabbit anti-bPL IgG into the fetal catheter. Broken line arrow indicates the day of calving.

**Figure 3:**
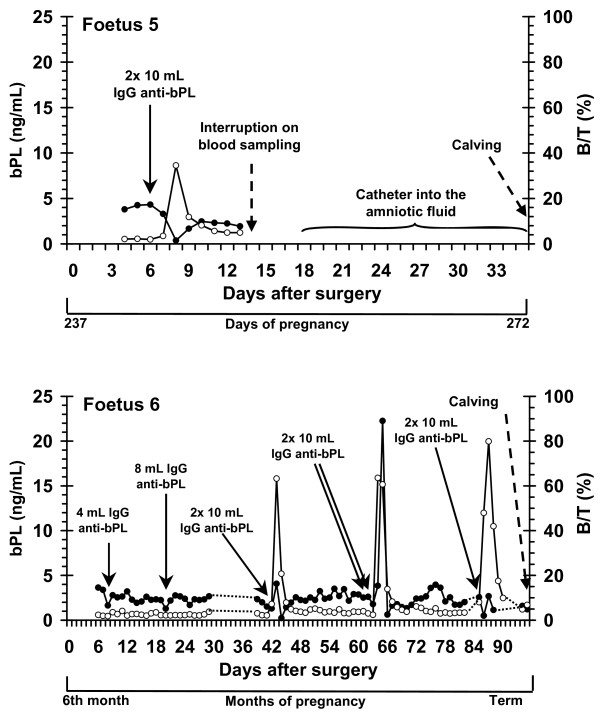
**Plasmatic profiles of bPL concentrations and rabbit anti-bPL titers in peripheral circulation of two bovine foetuses**. Concentrations of bPL in fetal plasma (ng/mL) are represented by black dots. Anti-bPL titers measured as B/T (bound activity (B) regarding total tracer (T) added) are represented by white circles. Plasma samples from cannulated foetuses (Foetus 5 and 6) were obtained during late pregnancy. Concentrations of bPL were measured by RIA with guinea pig anti-bPL antiserum as the primary antibody. Solid line arrows indicate day of infusion of a pool of rabbit anti-bPL IgG into the fetal catheter. Broken line arrow indicates the day of calving.

Figures 2 and 3 show the bPL concentrations as well as the binding activity (B/T) of the infused rabbit anti-bPL IgG measured in 6 cannulated foetuses during late pregnancy.

Catheter of foetuses remained functional for the longest period (95 days) in Foetus 6, despite a brief interruption in sampling between Days 30 and 38 (Figure 3). In Foetus 5 (Figure 3), catheter allowed blood sampling only during 10 days (Days 4 to 13). In this animal, after an interruption of 5 days in blood collection, the catheter was used to collect amniotic fluid during a 16-day period (data not shown). In the other 4 animals, catheter remained functional allowing blood sampling until 27 (Foetus 1) to 39 Days (Foetus 3) after the surgery.

Before IgG infusion, plasmatic concentrations of bPL ranged from 2.2 (Foetus 1) to 6.9 ng/mL (Foetus 2) at 236 and 239 days of pregnancy, respectively. Binding activity measured before IgG infusion (nonspecific binding) ranged from 1.9 (Foetus 6) to 4.6% (Foetus 4).

After a single injection of 8 mL of IgG in Foetus 1, bPL concentrations discreetly decreased from 1.9 to 1.1 ng/mL (Figure 2). In this foetus, concentrations of bPL remained relatively constant until parturition (range from 1.9 to 3.8 ng/mL). In Foetus 2, bPL concentrations decreased on the day following the IgG infusion into the foetal circulation. The day after, concentrations reached a peak, decreased and remained relatively constant until parturition. In Foetus 3, bPL concentrations also reached a peak two days after injection of IgG anti-bPL. Thereafter, concentrations tended to increase until parturition.

As detailed in Table 1, Foetus 4 received a succession of infusions of bPL at 9-12-hour interval (Day 7, 13 to 14 and 22 to 27 after surgery). Interestingly, in this foetus, bPL concentrations first decreased (Day 8) and thereafter increased until Days 13-14, when the next infusions were injected into the catheter. And then, bPL concentrations increase significantly to reach higher levels (14.0 ng/mL) at Day 25 after surgery. Just before parturition, concentrations of bPL decreased to reach 11.0 ng/mL.

In Foetus 5, concentrations of bPL were measured for a short time, decreasing to 0.5 ng/mL after IgG injection. In this animal, the catheter was stripped out of the blood vessel and it remained in the amniotic compartment from Day 18 onward (data not shown).

Finally, concentrations of bPL remained relatively constant in the peripheral circulation of Foetus 6 during the whole sampling period, despite successive injections of purified anti-bPL IgG. Binding activities immediately after IgG injections were comparable to those observed in Foetuses 2 to 5 (B/T higher than 60%).

## Discussion

Passive immunoneutralization of an endogenous factor associated with establishment of its secretion pattern via a frequent blood sampling constitutes a powerful tool for dissecting the contribution of that factor to normal endocrinological function [28]. Bovine placental lactogen, also known as bovine chorionic somatomammotropin, is believed to play a pivotal role in the growth and development of the foetus by coordinating the maternal metabolism and nutrient supply from the cow to the foetus [29]. The predicted secreted form of bPL has 200 residues and its primary sequence exhibits 50% and 23% homology to bovine prolactin (bPRL) and growth hormone (bGH), respectively [16,30]. Native 30-33 kDa bPL forms have been purified from the placenta of cows [20,31-34] and some of them were successfully used to raise antisera in rabbits [17,35]. In the present investigation, we described the use of a bPL-RIA system based on guinea pig antiserum for measurement of foetal bPL concentrations after immunoneutralization with rabbit anti-bPL antibodies. Moreover, we described for the first time a long-term foetal catheterization allowing following up the changes in bPL concentration after injection of purified anti-bPL IgG into foetal compartment.

Most of the studies describing the interference of antibodies with immunoassay measurements were carried out in human medicine concerning serum thyroglobulin measurements in the presence of thyroglobulin auto-antibodies [36,37]. Many endocrinologists were also confronted with this problem when investigating diabetes mechanism after administration of exogenous insulin antiserum [38,39] or when investigating the physiological role of oPL following active immunization of ewe-lambs against recombinant oPL [40]. As stated by Schneider and Pervos [41], the magnitude and direction of interference of endogenous or exogenous antibody are determined by the affinity of the first antibody, the species specificity of the second antibody, and the volume of the serum used, among others. In the present study, the use of a primary guinea pig anti-bPL antiserum appropriately quantified bPL concentrations in peripheral concentration of nonimmunized foetuses (concentrations ranging from 6.72 to 22.56 ng/mL). The range of bPL concentrations was in agreement with previous findings with regards to bovine foetuses by the use of rabbit anti-bPL antiserum [17,35,42]. Our results also showed that rabbit anti-guinea pig secondary antibody was more specific than sheep anti-rabbit antibody for the recognition of primary antisera. However, in the *in vitro *study, when rabbit primary antiserum was added at dilutions lower than at 1:20,000, the recovery of bPL by use of guinea pig primary antiserum decreased significantly (<80%). So, measurement of bPL concentrations in the presence of exogenous rabbit anti-bPL by using guinea pig anti-bPL primary antiserum can reduce but does not eliminate completely the interference of exogenous antibodies when present in higher titers. Moreover, as observed in Table 3, high circulating antibody titers led to a higher interference with the recovery of the added amount of bPL (12 ng/mL). We suggest a threshold exogenous anti-bPL level (titer 1:20,000 to 1:40,000) below which interference can be expected.

During the past decades, foetal catheterization in the large domestic animal species has proven to be an important tool that contributed for the determination of foetal hormonal profiles and for following up the changes in the peripheral hormonal circulation after imunomodulation bioassays. As early as in 1974, Comline et al. [43] studied the hormonal changes associated with the artificial induction of labor in bovine foetuses (240-260 days of gestation) by applying this technique to inject cortisol, dexamethasone, and corticotrophin to foetal circulation as well as to take blood samples during a period of 20 days. In our study, foetal blood samples could be successfully obtained during a long period (10 to 95 days) after cannulation surgery. This sampling period throughout late gestation was much longer than those reported in the literature from ovine (3 days [5]; 10 days [44]; 14 days [7]; 35 days [45]) and bovine (4 days [46]; 15 days [26]; 24 days [47]) foetuses.

The use of passive immunoneutralization technique in order to abolish an endogenous factor by using a specific antisera predates the discovery that pituitary hormone secretion is pulsatile in nature [28]. This method was used in studies on the endocrine function of several hormones such as insulin [48], glucagon [49,50], luteinizing hormone [51], and insulin-like growth factor-I [52,53]. In order to investigate the physiological role of placental lactogen, Waters et al. [8] infused ewes during late gestation with goat anti-oPL antiserum in order to neutralize oPL for at least 12 h. In their study, as well as in ours, the potential interference of the infused antiserum with the RIA measurement was taken into consideration. These authors used an antiserum generated in a species other than that used to raise the RIA's primary antiserum (rabbit anti-bPL).

Studies on foetal growth endocrinology using foetal cannulation technique were more frequently carried out in ovine than in bovine species for obvious reasons (cost, duration of pregnancy, accessibility to foetal compartment, housing structures, and others) [54]. However, due to the intrinsic characteristic of oPL and bPL hormones, it cannot be assumed that the results obtained in the ovine model are adequate for better understanding of PL physiology in the cow. As previously described, while the placental oPL is almost secreted entirely into the dam (with the foetal levels being 100-fold lower) [55], in cows the bPL concentrations are higher in foetal than in maternal compartments until parturition [17]. Moreover, maternal concentrations of oPL increase from 100 to 1,000 ng/mL between Days 70 and 130 of gestation [56], whereas maternal concentrations of bPL remain under 2 ng/mL during the whole pregnancy. Finally, oPL is a nonglycosylated protein, whereas bPL is a glycosylated molecule.

The plasma levels of placental products are regulated by the overall rate of biosynthesis at the source level, utilization at the target tissue(s) level, and clearance from the circulation. In foetuses 3 and 4, following injection of purified anti-bPL IgG, concentrations of bPL tended to increase in foetal circulation, which resembles the enhancement of *in vivo *GH activity by anti-GH antibodies [57-59]. The precise mechanism by which anti-bPL antibodies enhance bPL concentrations is not clear. Short half-lives were estimated for some PL molecules, approximately 10.5 min and 7.5 min for [^125^I]oPL and rbPL, respectively [16]. However, considering that native bPL is a glycosylated molecule, half-life may be probably longer than that of rbPL. Another possible explanation could be that the complex formed by the infused anti-bPL IgG antibodies and free bPL protects this molecule from the degradation, prolonging its half-life. It is also possible that anti-bPL may induce changes in the molecular structure of bPL, increasing its affinity for its receptor or decreasing hormone-receptor internalization rate. Enzymatic removal of N-linked oligosaccharide from bPL increased the affinity for its receptor by approximately two-fold [16]. An alternative explanation for the increase in the bPL concentrations in peripheral circulation of Foetuses 3 and 4 is that the immunoneutralization of bPL activity led to an increase in bPL synthesis and secretion by the placenta through an altered feedback mechanism, as a compensatory "rebound effect".

In the present *in vivo *study, detection of rabbit anti-bPL IgG was possible in all the infused foetuses. The attained titers (dilutions giving up to 50% of specific antibody binding) were comparable to those values and to the variability of responses reported after immunization with hormones such oPL [40]. The infusion of anti-bPL IgG immediately immunoneutralized the circulating bPL, as reported by Waters et al. [8]. Before the infusion of anti-bPL, basal concentrations of bPL are in accordance with those reported by different authors [17,22]. After bPL immunoneutralization, a rapid decline of anti-bPL was observed, alleviating the neutralization effect. This decrease is probably due to the combined effect of degradation, clearance from the circulation, and filling of available binding sites with endogenous bPL.

In rat, responses to GH have been shown to vary depending on the pattern of GH administration. GH injections have a more pronounced effect on total body weight gain, whereas a constant infusion of GH leads to selective organ growth and reduction in size of fat pads [60]. As seen in Table 1, the infusions were made occasionally; we did not use any device to infuse anti-bPL continuously for a long period.

In summary, our data demonstrated the feasibility and utility of a bPL-specific assay using a guinea pig anti-bPL antiserum in investigations based on neutralization of circulating bPL by means of direct injection of rabbit immunoglobulins into the foetal circulation. In addition, long-term foetal catheterization in late gestation has proven to be realizable and can be proposed as a tool to investigate foetal endocrinology during late pregnancy.

## Competing interests

The authors declare that they have no competing interests.

## Authors' contributions

AVAO carried out all radioimmunoassays, assisted in surgical procedure and in blood sampling, carried out the analysis of data and drafted the manuscript. NMS participated in the design of the study, performed pre- and post-surgical care, assisted in surgical procedure, carried out blood sample collection, has been involved in interpretation of data and revised the manuscript critically for intellectual content. JLH assisted in surgical procedure and performed the statistical analysis. KT performed surgical procedure and participated in pregnancy follow-up until calving. GCVDW gave critical advice for the elaboration of the protocol of foetal cannulation and performed surgical procedure. MAMT gave critical advice for the elaboration of the protocol of foetal cannulation and coordinated different steps of surgical procedure. OS performed surgical procedure. JFB conceived the study, coordinated all different parts of the experimental design, participated in analysis of data and performed critical revision of the manuscript for important intellectual content. All authors read and approved the final version of the manuscript.
